# Supercritical Fluid Chromatography—Tandem Mass Spectrometry for Rapid Quantification of Pentacyclic Triterpenoids in Plant Extracts

**DOI:** 10.3390/ph15050629

**Published:** 2022-05-20

**Authors:** Danil I. Falev, Denis V. Ovchinnikov, Ilya S. Voronov, Anna V. Faleva, Nikolay V. Ul’yanovskii, Dmitry S. Kosyakov

**Affiliations:** Laboratory of Natural Compounds Chemistry and Bioanalytics, Core Facility Center “Arktika”, M.V. Lomonosov Northern (Arctic) Federal University, Northern Dvina Emb. 17, 163002 Arkhangelsk, Russia; d.ovchinnikov@narfu.ru (D.V.O.); i.voronov@narfu.ru (I.S.V.); a.bezumova@narfu.ru (A.V.F.); n.ulyanovsky@narfu.ru (N.V.U.)

**Keywords:** pentacyclic triterpenoids, plant feedstock, supercritical fluid chromatography, tandem mass spectrometry

## Abstract

Pentacyclic triterpenoids (PCTs) are a widely distributed class of plant secondary metabolites. These compounds have high bioactive properties, primarily antitumor and antioxidant activity. In this study, a method was developed for the quantitative analysis of pentacyclic triterpenoids in plants using supercritical fluid chromatography–tandem mass spectrometry (SFC-MS/MS). Separation of ten major PCTs (friedelin, lupeol, β-amyrin, α-amyrin, betulin, erythrodiol, uvaol, betulinic, oleanolic and ursolic acids) was studied on six silica-based reversed stationary phases. The best results (7 min analysis time in isocratic elution mode) were achieved on an HSS C18 SB stationary phase using carbon dioxide—isopropanol (8%) mobile phase providing decisive contribution of polar interactions to the retention of analytes. It was shown that the use of atmospheric pressure chemical ionization (APCI) is preferred over atmospheric pressure photoionization (APPI). The combination of SFC with APCI-MS/MS mass spectrometry made it possible to achieve the limits of quantification in plant extracts in the range of 2.3–20 μg·L^−1^. The developed method was validated and tested in the analyses of birch outer layer (*Betula pendula*) bark, and licorice (*Glycyrrhiza glabra*) root, as well as lingonberry (*Vaccinium vitis-idaea*), cranberry (*Vaccinium oxycoccos*), apple (*Malus domestica* “*Golden Delicious*” and *Malus domestica* “*Red Delicious*”) peels.

## 1. Introduction

Pentacyclic triterpenoids (PCTs) are a widely distributed class of important plant secondary metabolites. The most abundant triterpenoids in higher plants are derivatives of pentacyclic hydrocarbons lupane, oleanane and ursane, which contain 30 carbon atoms and belong to the monool, diol, ketone and triterpenic acid types [1,2], differing significantly in polarity and solubility. PCTs possess various types of bioactivities, having anti-inflammatory, antimicrobial, antihyperlipidemic, hepatoprotective, gastroprotective, antidiabetic and hemolytic properties, as well as cardiovascular, antipyretic and wound healing effects [3,4,5,6,7,8,9,10]. Many researchers emphasize the antitumor [3,4,10,11,12,13,14] and antioxidant activities of PCTs [6,7,15,16], which have also been observed in triterpenoid-rich plant extracts [17,18,19,20,21,22,23]. Therefore, PCTs are of exceptional interest for the pharmaceutical, cosmetic and food industries. Thus, the development of new rapid, selective, and highly sensitive methods for screening and determination of triterpenoids in plant materials, drugs, and biological fluids is an important task.

Mass spectrometry techniques, especially MALDI MS, have been successfully used for the rapid screening of triterpenoids in plant materials [24], while the quantitative analysis of PCTs requires chromatographic separation of analytes. High-performance liquid chromatography–tandem mass spectrometry (HPLC-MS/MS) in multiple reaction monitoring (MRM) mode is widely used for PCT quantitative analysis [25,26,27,28]. The authors have found that it is preferable to use atmospheric pressure chemical ionization (APCI) with positive ion mode, whereas using electrospray ionization (ESI) causes a decrease in PCT ionization efficiency due to the low polarity of the analytes (except triterpenic acids in negative ion mode). 

Since different types of triterpenoids strongly differ from each other in terms of polarity, and each type includes structurally similar compounds with close retention on the common HPLC stationary phases, the chromatographic separation of complex mixtures of PCTs is a nontrivial task. It requires gradient elution and long analysis time when using reversed stationary phases which, however, do not provide a good chromatographic resolution between similar compounds with the same monoisotopic mass in some critical pairs of PCTs, such as erythrodiol/uvaol, β-amyrin/α-amyrin, and oleanolic/ursolic acids. Using the stationary phase with cross-linked octadecyl groups (Nucleodur C18 Isis) with higher resolution towards positional isomers made it possible to separate nine PCTs in gradient elution mode in only 25 min and achieve the limits of quantitation (LOQ) in HPLC-MS/MS analysis in the range of 10–22 μg·L^–1^ [27]. Stationary phases with a mixed retention mechanism can be used to reduce analysis time and enhance selectivity. In our recent study [28], the application of the Acclaim Mixed-Mode WAX-1 stationary phase combining ion exchange, hydrophilic interactions, and reversed phase retention in isocratic mode allowed rapid separation of ten PCTs (including mentioned above critical pairs) in 7 min with LOQs of 8.3–150 μg·L^–1^. The advantage of the method is the ability to fine tune the separation selectivity by changing the contributions of various types of interactions to the retention mechanism with a change in the composition and pH of the mobile phase. On the other hand, due to the many factors affecting separation, the development and optimization of an analytical method for such stationary phases requires considerable effort. 

A modern and “greener” alternative to HPLC in the analysis of complex mixtures is supercritical fluid chromatography (SFC) using sub- or supercritical carbon dioxide as a main component of the mobile phase. Due to the low viscosity and thus high diffusion coefficients of such media, SFC features high mass transfer rate, higher efficiency, and exceptional separation speed. It has been successfully used for rapid analysis of various natural compounds [29,30,31,32]. There are few published works on the use of SFC for the analysis of PCTs; the most recent one [33] uses a combination of SFC with light scattering detection (ELSD). Although separation of eight PCTs was achieved in isocratic mode within 16 min, the drawback of this approach is low sensitivity and selectivity of ELSD. Considering the possibility of rapid separation of PCTs by the SFC, it is promising to hyphenate it with sensitive tandem mass spectrometric detection, combining the advantages of these separation and detection techniques. Thus, the aim of the present study is to develop a novel rapid and sensitive method for the simultaneous determination of pentacyclic triterpenoids in plant biomass by SFC-MS/MS. 

## 2. Results and Discussion

### 2.1. Tandem Mass Spectrometry

**Figure 1:**
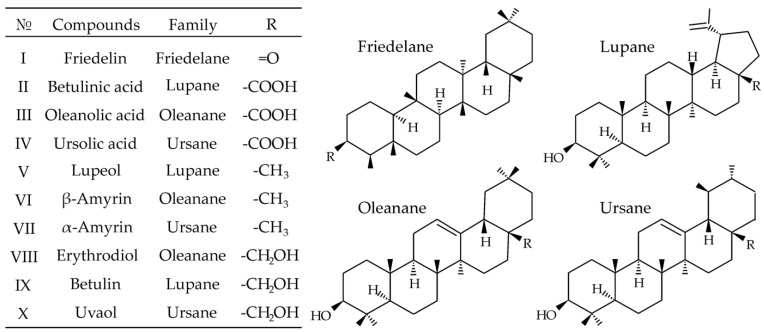
Chemical structure of the studied pentacyclic triterpenoids.

The ten most common representatives of monools (lupeol, α- and β-amyrins), diols (erythrodiol, betulin, uvaol), ketones (friedelin) and triterpenic acids (betulinic, oleanolic, ursolic) with friedelane, lupane, oleanane and ursane backbones (Figure 1) were chosen as target analytes. As can be seen from their structures, PCTs are characterized by rather low polarity, which makes APCI or atmospheric pressure photoionization (APPI) preferable for the mass spectrometric detection. Even though triterpenic acids can form deprotonated molecules under SFC-APCI/APPI conditions, the positive ion mode demonstrated an undoubted advantage, allowing efficient ionization of all selected analytes, including those containing no acidic groups. It has been established that all hydroxyl-containing analytes undergo dehydration in the ion source during APCI/APPI. Thus, they form [M + H − H_2_O]^+^ species, whereas friedelin gives protonated molecule [M + H]^+^. These ions were chosen as precursor ions in tandem mass spectrometry. 

The optimization of APCI and APPI parameters was carried out in selected ion monitoring mode by flow injection of analyte standard solutions into the ion source with a flow of SFC mobile phase to achieve maximum intensities of the signals of precursor ions. The obtained optimal conditions were as follows: APCI: ion source temperature—350 °C; curtain gas, nebulizer gas and drying gas pressure—25, 20 and 30 psi, respectively; nebulizer current—3.5 µA; APPI: ion source temperature—350 °C; curtain gas, nebulizer gas and drying gas pressure—10, 20 and 30 psi, respectively; ion spray voltage—750 V. After obtaining tandem mass spectra, the most intense ion transitions were chosen and the optimal parameters (declustering and entrance potentials, collision energy) for detecting PCTs in MRM mode were determined (Table 1).

### 2.2. Chromatographic Separation and Column Screening

Preliminary tests with BEH Silica and 2-ethylpyridine polar stationary phases showed high retention of most polar analytes, especially triterpenic acids, along with unacceptable chromatographic peak shapes. For this reason, the present study was focused on the use of six silica-based reversed stationary phases—Accucore C30, Nucleodur C18 Isis, Luna C18, Zorbax SB-Aq, HSS C18 SB and Nucleodur PolarTec. The latter is distinguished by the presence of amide groups embedded into alkyl chain and contributing to the retention mechanism through polar interactions. SFC column screening was carried out using the model mixture of analytes under the following basic conditions: temperature of 25 °C, backpressure 150 bar, and eluent flow rate 1.0 mL·min^–1^. The mobile phase was 90% carbon dioxide and 10% methanol. 

Despite the similarity of the tested stationary phases, the obtained chromatograms (Supplementary Materials, Figure S1) demonstrated significant differences in retention, selectivity and elution order of the analytes. As expected, triacontyl (C30) and cross-linked octadecyl (C18 Isis) phases are characterized by similar patterns of retention, with elution order corresponding to the decrease in polarity of analytes: triterpenic acids–diols–monools. This indicates the decisive contribution of the reversed phase (hydrophobic) interactions into the mechanism of analyte retention. The only difference between two phases is the position of the peak of friedelin, which has a higher retention on the C30 column. Unacceptably low retention factors (*k*) and thus the impossibility of separating the most polar compounds (diols and triterpenic acids) make such stationary phases unsuitable for solving the problem of PCTs analysis. This also applies to the Zorbax SB-Aq stationary phase, in which the polar surface of the silica particles apparently contributes to the retention (higher *k* values for diols) but is difficult to access for the analytes due to steric hindrances caused by the presence of isopropyl side chains in the grafted alkyl groups.

A completely different pattern was observed for non-endcapped stationary phases with residual silanol groups capable of interactions with analytes (Luna C18, HSS C18 SB), as well as for a Nucleodur PolarTec sorbent. They provide much higher *k* values for all studied PCTs due to mixed-mode retention involving both hydrophobic and polar interactions. Despite this, at a relatively high concentration of methanol (moderately polar mobile phase) the contribution of the reversed phase mechanism is decisive, and the order of analytes elution on both octadecyl phases does not differ from that of the triacontyl sorbent. Due to the greater availability and different nature of the polar groups in the Nucleodur PolarTec phase, the retention of the most polar analytes on this sorbent increases significantly and the separation selectivity is unacceptable. In this regard, based on the results of column screening, the stationary phase HSS C18 SB, which was especially designed to increase the selectivity of the separation of substances with polar groups in SFC and showed the best separation of the investigated PCTs, was chosen for further optimization of the chromatographic method.

Variations of temperature (20–55 °C) and backpressure (110–190 bar) did not significantly affect the separation of triterpenoids on an HSS C18 SB column, which is in good agreement with the published data on the minority of these parameters when optimizing separation in SFC on polar stationary phases [31]. To ensure the maximum chromatographic column lifetime and the stability of maintaining the temperature, we chose 25 °C as the optimal value. The backpressure of 150 bar typical for SFC separations was used in our further work.

**Figure 2:**
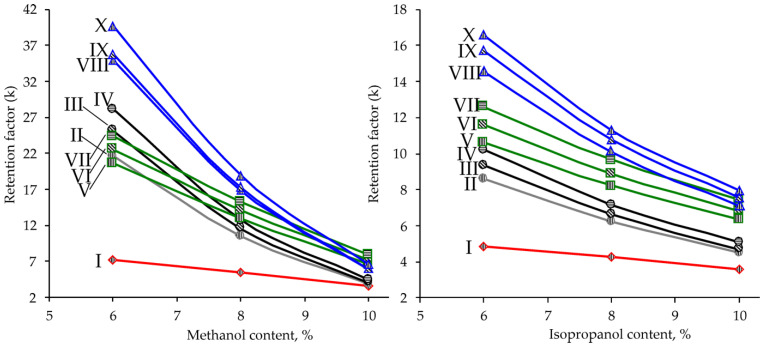
Effect of methanol (**left**) and isopropanol (**right**) content in the mobile phase on the retention factors of PCTs on HSS C18 SB stationary phase (flow rate 1.5 mL·min^–1^, T = 25 °C, backpressure 150 bar).

The main parameter affecting the retention and selectivity in SFC is organic modifier (co-solvent) content in the carbon dioxide-based mobile phase. A decrease in the methanol concentration from 10 to 6% led to a sharp (2–4-fold) increase in the *k* values (Figure 2) for all analytes, except for friedelin, which does not possess hydroxyl or carboxyl groups. The most important feature of the obtained dependences of the retention factors on the methanol content is the inversion of the elution order of PCTs belonging to the monools and triterpenic acids (in the range of 6–8% MeOH), as well as monools and diols (in the range of 8–10% MeOH). Thus, at methanol concentrations ≤ 6%, this leads to the occurring the elution order (friedelin–monools–acids–diols) characteristic of retention by the mechanism, the main contribution to which is made by polar interactions of analytes with silanol groups of the sorbent. Varying the methanol concentration leads to a significant change in the contributions of hydrophobic and polar interactions to the mixed retention mechanism of PCTs with the possibility of transition from reversed-phase separation to the normal-phase one, and vice versa. The reason for this phenomenon is the competition of analytes and polar mobile phase modifier for sorption centers (silanol groups) of the stationary phase. As can be seen, achieving complete separation of all analytes requires a methanol concentration of <6%, which is irrational both from the point of view of an unacceptably high retention of diols (*k* > 40) and a decrease in the solubility of PCTs in the mobile phase with the risk of precipitation and clogging the chromatographic tract. To overcome this problem, methanol as a mobile phase modifier was replaced by the less polar isopropanol. 

The obtained results (Figure 2) show that the lower affinity of isopropanol with respect to the silica surface makes it possible to implement a predominantly normal-phase separation mechanism in the entire studied concentration range. Sufficiently complete separation of analytes was achieved at a modifier content of 6–8%, while providing optimal values of retention factors in the range of 4–17. To ensure the maximum separation rate, the working concentration of isopropanol in the mobile phase of 8% and increased eluent flow rate of 1.5 mL·min^–1^ were chosen for analytical method development.

**Figure 3:**
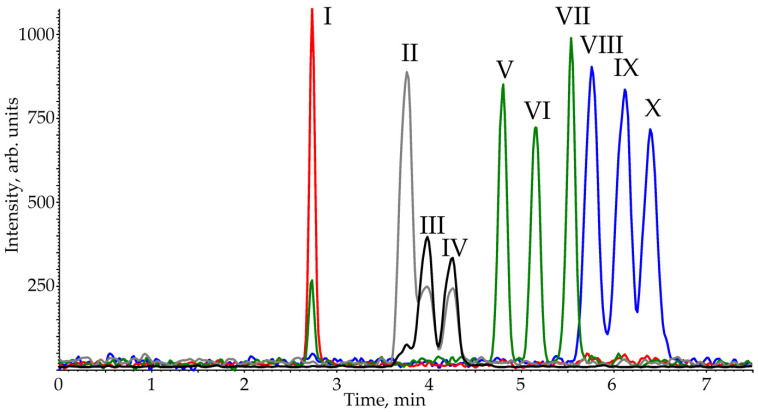
SFC-APCI-MS/MS chromatogram of analytes model mixture (I—200 μg·L^–1^; II, VIII and X—100 μg·L^–1^; III, IV and IX—50 μg·L^–1^; V, VI and VII—25 μg·L^–1^) obtained on HSS C18 SB stationary phase in the optimized conditions.

Thus, the optimal separation parameters of ten PCTs are: HSS C18 SB stationary phase, 92% carbon dioxide and 8% isopropanol mobile phase, elution rate—1.5 mL·min^–1^, a temperature—25 °C, and backpressure—150 bar. The SFC-APCI-MS/MS chromatogram obtained under these conditions is presented in Figure 3.

This demonstrates that the analysis time (7 min) is 2–3 times lower than those reported in the literature for reversed phase HPLC [27] or even SFC on polar sorbent [33] methods and is comparable to the value attained in our recent work using HPLC on a stationary phase with mixed retention mechanisms [28]. It is worth noting that SFC, unlike mixed mode HPLC, does not require complex and time-consuming method optimization procedures due to there being fewer parameters affecting the separation. Despite the short retention times, a baseline separation was observed for most PCTs (Table S1). The minimum chromatographic resolution (*Rs*) was 0.83 for the betulinic/oleanolic acid critical pair. However, these compounds are distinguished with different ion transitions in MRM detection. The *Rs* values for other pairs of analytes (oleanolic/urosolic acids and betulin/uvaol) were close to 1.0; however, this does not create significant problems for the quantitative determination of these PCTs.

### 2.3. Quantification and Method Validation

Since the LOQs of triterpenoids obtained using APCI (2.3–20 μg·L^–1^) were somewhat lower than those attained in APPI-MS/MS (3.5–33 μg·L^–1^) (Table 2), the APCI method was used for further experiments. The reliability of the obtained LODs and LOQs was proved by the analysis of a model mixture of analytes with concentration close to the limit of quantitation (Figure S2). The developed method demonstrated a high sensitivity comparable to that reported in previous works using HPLC-MS/MS (8.3–150 μg·L^–1^) [27,28], even exceeding it. The calibration dependences were linear (R^2^ > 0.999) for all ten triterpenoids (from LOQ to the maximum used concentrations), spanning at least two orders of magnitude (Table 2).

The attained accuracy was close to 100%, and the standard deviation did not exceed 10% in intra-day and inter-day assays (Table S2) at levels close to LOQ.

The matrix effects assessment was carried out by the spike recovery test using the licorice root PLE extract as a real matrix. The recovery values were in the range of 88–118%, which proved the absence of significant interferences from the matrix for all analytes (Table S3). The efficient elimination of the matrix effect is due to the high sensitivity of the method, allowing sample dilution, the use of APCI not highly susceptible to interference from other components, and chromatographic separation of the triterpenoids from the matrix.

The use of isocratic elution and optimal conditions of the chromatographic separation of analytes ensure the robustness of the developed approach. Variations in pressure (145–155 bar) and temperature (23–27 °C) did not lead to a significant (>2%) shift in *t_R_* values and loss of chromatographic resolution *Rs*. The chromatographic separation did not deteriorate after repeated analysis of plant extracts (about 100 injections).

### 2.4. Plant Biomass Analyses

**Figure 4:**
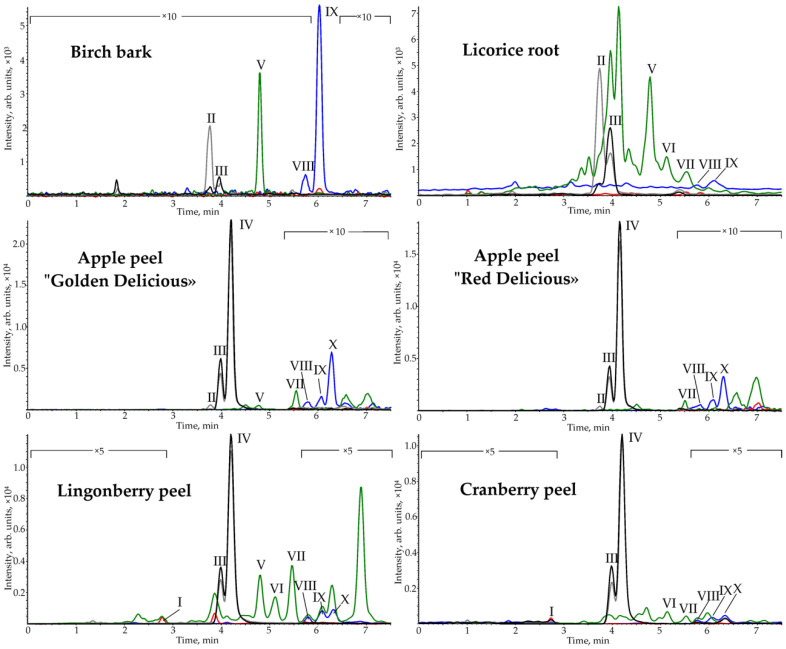
SFC-APCI-MS/MS chromatograms of plant methanolic PLE extracts on HSS C18 SB stationary phase.

To test the developed method, licorice root, birch bark, and berry and apple peels were selected as real objects. These objects are characterized by a complex chemical composition and are considered an important source of biologically active substances for pharmaceutical, cosmetic and food purposes. PLE with methanol, providing near quantitative extraction of PCTs, was used as a sample preparation method [26]. The resulting chromatograms (Figure 4) demonstrate the presence of all analytes in the samples (Table 3) over a wide range of concentrations (0.0041–250 mg·g^−1^).

The outer layer of birch bark is dominated by betulin, erythrodiol, uvaol, betulinic and oleanolic acids (3.6–250 mg·g^−1^). The content of the components is consistent with the literature data [27,28].

Betulinic acid predominates in licorice root extract. Minor substances (0.0041–0.1 mg·g^–1^) are oleanolic acid, lupeol, β-amyrin, α-amyrin, erythrodiol and betulin. Most triterpenoids in licorice root have been previously reported [27,34,35].

Major triterpenoids of apple peel are betulinic, oleanolic and ursolic acids, as well as uvaol. At the same time, the content of triterpene acids in the peel of “Golden Delicious” apples is higher compared to “Red Delicious”. Minor components (0.027–0.26 mg·g^–1^) are lupeol, α-amyrin, betulin and erythrodiol. The presence of most components in apples has been previously reported [27,36,37,38]. However, the papers [37,38] reported only the sum content of the erythrodiol and uvaol, since it was not possible to separate this pair of substances.

Berry peel extracts differ from apple peel in the presence of the ketone friedelin. The main difference between lingonberry peel and cranberry peel is the increased content of monools. Their total content in the lingonberry peel is 6-fold higher than in the cranberry peel. The major components of berry peels are ursolic and oleanolic acids (3.2–15 mg·g^–1^). The minor components of berries peel are diols. The presence of main triterpenoids has been reported previously [27,28,39,40,41].

## 3. Materials and Methods

### 3.1. Reagents and Materials

Commercially available standards of ten studied pentacyclic triterpenoids—friedelin (tech. grade), betulinic acid (≥97.0%), oleanolic acid (≥97.0%), ursolic acid (≥90.0%), lupeol (≥90.0%), β-amyrin (≥98.5%), α-amyrin (≥98.5%), erythrodiol (≥97.0%), betulin (≥98.0%), and uvaol (≥95.0%)—were purchased from Sigma-Aldrich (Steinheim, Germany).

HPLC gradient grade methanol and isopropanol (Chimmed, Moscow, Russia), and carbon dioxide (≥99.99%, Kriogen, Moscow, Russia) were used for the preparation of mobile phase. Methanol was also used for analyte solution preparation and PLE extraction of plant materials. 

The stock solutions of triterpenoids in methanol (250 mg·L^−1^) were prepared from an accurate sample. Calibration solutions of analytes were prepared by mixing and successive dilutions of the stock solutions with methanol. The solutions were stored in the dark at 4 °C for no more than one week. Stability was checked once a day.

### 3.2. Plant Materials and Extraction

The silver birch (*Betula pendula*) bark, cranberry (*Vaccinium oxycoccos*) and lingonberry (*Vaccinium vitis-idaea*) fruits were collected in the forests of the Arkhangelsk region of Russia in August 2021. Identification of botanical raw materials was carried out according to the herbarium of Northern (Arctic) Federal University. The apple fruits (*Malus domestica* var. *Golden Delicious* and *Malus domestica* var. *Red Delicious*) and licorice (*Glycyrrhiza glabra*) roots were purchased in the retail chain in August 2021. The outer layers of bark, root, and berry and apple peels were separated manually and dried in oven at 50 °C overnight. Dry plant material was grinded (0.5–1 mm) and stored in desiccator over silica gel in dark at room temperature.

Pressurized liquid extraction (PLE) was performed on an ASE-350 accelerated solvent extraction system (Dionex, Sunnyvale, CA, USA) according to a previously developed method [26]. A sample of dry plant material (1.0 g) was extracted with methanol (two extraction cycles of 10 min each) at 100 °C and 100 bar under nitrogen. The resulting extract was dried. The dry extract (1.00 mg) was dissolved in 1000 μL of methanol, dilute with methanol, filtered through a nylon membrane filter (0.22 μm) and subjected to chromatographic analysis.

### 3.3. Supercritical Fluid Chromatography and Mass Spectrometry

The SFC-MS/MS system was used, which consisted of a 3200 QTrap triple quadrupole mass spectrometer (ABSciex, Vaughan, ON, Canada) equipped with APCI (Turbo-V) and APPI (Photospray) ion sources, and an Acquity UPC^2^ SFC system (Waters, Milford, MA, USA), including four pumps for supplying carbon dioxide and co-solvent, autosampler, column thermostat, and backpressure regulator. Make-up solvent and dopant (in APPI) were pumped using an additional Ultimate 3000 RS HPLC system (Thermo Scientific, Waltham, MA, USA).

Separation was carried out on the following columns: Accucore C30 150 × 2.1 mm, 2.6 µm (Thermo Scientific, Waltham, MA, USA), Luna C18 250 × 4.6 mm, 5.0 µm (Phenomenex, Torrance, CA, USA), Nucleodur C18 Isis 150 × 2.0 mm, 1.8 µm (Macherey-Nagel, Duren, Germany), Nucleodur PolarTec 150 × 3.0 mm, 3.0 µm (Macherey-Nagel, Duren, Germany), Zorbax SB-Aq 150 × 3.0 mm, 3.5 µm (Agilent, Santa Clara, CA, USA) and Acquity UPC^2^ HSS C18 SB 150 × 3.0 mm, 1.8 µm (Waters, Milford, MA, USA). The volume of the injected sample was 5 µL. Flow rate of make-up solvent (isopropanol) was 0.1 mL·min^−1^. The SFC-MS/MS system was controlled, and data were collected and processed using the Empower 3.0 software package (Waters, Milford, MA, USA).

The parameters of the APCI and APPI ionization sources varied in the ranges 250–450 °C for the temperature of the ion source; 10–20, 10–30, and 10–40 psi for curtain gas, nebulizer gas, and dryer gas, respectively; 2.5–5 mA for nebulizer current (APCI); and 650–1000 V for ion spray voltage (APPI). The parameters of the MRM mode varied in the ranges 10–100 V for declustering potential and collision energy and 1–12 V for entrance potential. Toluene (0.1 mL·min^–1^) was used as a dopant in APPI. 

Optimization of PCT separation was carried out by APCI-MS using the SIM (single ion monitoring) mode: I—427 *m/z*; II, III and IV—439 *m/z*; V, VI and VII—409 *m/z*; VIII, IX and X—425 *m/z*. Optimization of the ionization source parameters was also carried out using the SIM mode.

### 3.4. Method Validation

The values of the lower limit of quantification (LOQ) of ten triterpenoids were determined using a signal-to-noise ratio (S/N) criterion of 10. The intra-day precision was estimated at the lowest concentration level (close to LOQ). A series of consecutive chromatographic analyses of the analytes (*n* = 7) was used. The inter-day precision was determined in the same manner within 48 h (*n* = 14). The matrix effect and accuracy of PCTs quantification in plant extracts were estimated by spike-recovery test. Three concentration levels of analytes were introduced into licorice root PLE extracts and analyzed (*n* = 3).

## 4. Conclusions

A fast, accurate, highly sensitive and green method for the analysis of pentacyclic triterpenoids of different types by supercritical fluid chromatography–tandem mass spectrometry with atmospheric pressure chemical ionization was developed and validated. The use of the HSS C18 SB stationary phase and isopropanol as mobile phase modifier allowed the rapid chromatographic separation of ten pentacyclic triterpenoids (friedelin, lupeol, β-amyrin, α-amyrin, betulin, erythrodiol, uvaol, betulinic, oleanolic and ursolic acids) with a mixed retention mechanism with prevailing polar interactions of analytes with silanol groups of the stationary phase. With an analysis time of 7 min, the developed method ensures LOQs in plant biomass extracts of 2.3–20 μg·L^–1^. The application of the developed method for the analysis of real objects—outer layer of birch (*Betula pendula*) bark, licorice (*Glycyrrhiza glabra*) root, and lingonberry (*Vaccinium vitis-idaea*), cranberry (*Vaccinium oxycoccos*), apple (*Malus domestica “Golden Delicious”* and *Malus domestica “Red Delicious”*) peels—allowed new data to be obtained on the contents of ten PCTs. The developed method can be used both for the analysis of plant materials, drugs, and biological fluids, as well as for pharmacodynamic and pharmacokinetic studies.

## Figures and Tables

**Table 1:** Optimized conditions of PCT mass spectrometric detection in MRM mode.

Analyte	Monoisotopic Mass, Da	Precursor Ion, *m/z*	Product Ion, *m/z*	Declustering Potential, V	Entrance Potential, V	Collision Energy, eV
I	426	427	95	41	4.5	45
II	456	439	95	61	5.5	45
III	456	439	191	43	5.0	19
IV	456	439	191	43	6.0	49
V	426	409	95	55	6.5	47
VI	426	409	95	55	8.0	49
VII	426	409	95	55	7.5	59
VIII	442	425	95	50	5.5	47
IX	442	425	95	50	6.0	19
X	442	425	95	50	7.5	21

**Table 2:** Calibration dependences (*y = a × x*) for the area of chromatographic peak versus analyte concentration, limits of quantification of analytes by SFC–APCI–MS/MS and SFC–APPI–MS/MS methods on HSS C18 SB stationary phase.

Analyte	APPI				APCI			
	Linear Concentration Range, μg·L^−1^	*a*	R^2^	LOQ, μg·L^−1^	Linear Concentration Range, μg·L^−1^	*a*	R^2^	LOQ, μg·L^−1^
I	33–2000	6.163	0.99288	33	20–2000	26.076	0.99945	20
II	7.0–2000	50.235	0.99278	7.0	11–2000	87.018	0.99997	11
III	3.5–1000	42.627	0.99422	3.5	4.0–1000	75.104	0.99997	4.0
IV	4.6–1000	38.455	0.99033	4.6	4.7–1000	68.122	0.99994	4.7
V	3.8–1000	139.33	0.99283	3.8	2.6–1000	219.15	0.99998	2.6
VI	4.5–1000	119.78	0.99833	4.5	2.7–1000	211.91	0.99997	2.7
VII	3.8–1000	147.89	0.99243	3.8	2.3–1000	253.00	0.99996	2.3
VIII	20–2000	35.314	0.99206	20	9.8–2000	92.152	0.99995	9.8
IX	11–1000	68.244	0.99116	11	5.5–1000	175.25	0.99997	5.5
X	27–2000	27.677	0.99410	27	13–2000	75.618	0.99993	13

**Table 3:** The content of PCTs (mg·g^–1^, recalculated for the oven-dried plant material) in plant tissues (*n* = 3, *p* = 0.95).

Analyte	Birch Bark	Licorice Root	Apple Peel “Golden Delicious”	Apple Peel “Red Delicious”	Lingonberry Peel	Cranberry Peel
I	-	-	-	-	0.17 ± 0.01	0.14 ± 0.01
II	13 ± 1	0.16 ± 0.01	0.79 ± 0.01	0.47 ± 0.02	-	-
III	3.6 ± 0.5	0.10 ± 0.01	10 ± 1	6.3 ± 0.5	3.5 ± 0.3	3.2 ± 0.2
IV	-	-	49 ± 5	32 ± 1	15 ± 2	14 ± 1
V	4.6 ± 0.3	0.043 ± 0.001	0.26 ± 0.02	-	0.80 ± 0.01	-
VI	-	0.0076 ± 0.0008	-	-	0.58 ± 0.01	0.23 ± 0.01
VII	-	0.0062 ± 0.0001	0.088 ± 0.006	0.027 ± 0.001	0.84 ± 0.01	0.12 ± 0.01
VIII	2.9 ± 0.1	0.0041 ± 0.0001	0.16 ± 0.01	0.090 ± 0.008	0.058 ± 0.003	0.030 ± 0.003
IX	250 ± 10	0.0073 ± 0.0002	0.12 ± 0.02	0.077 ± 0.002	0.072 ± 0.004	0.022 ± 0.002
X	-	-	1.1 ± 0.1	0.48 ± 0.05	0.17 ± 0.01	0.096 ± 0.007

## Data Availability

The data is contained in the article and Supplementary Materials.

## Supplementary Materials

The following supporting information can be downloaded at: https://www.mdpi.com/article/10.3390/ph15050629/s1, Table S1: Chromatographic separation parameters of ten PCTs on the stationary phase HSS C18 SB under optimal conditions. Table S2: The results of the evaluation of intraday and inter-day reproducibility analysis of the PCTs on HSS C18 SB stationary phase. Table S3: Matrix effect on the determination of PCTs on HSS C18 SB stationary phase. Figure S1: SFC-APCI-MS chromatogram (SIM mode) of analytes model mixture (250 μg·L^–1^ of each compound) obtained on various stationary phases. Figure S2: SFC-APCI-MS/MS chromatogram of analytes model mixture (I—25 μg·L^–1^; II, VIII and X—12.5 μg·L^–1^; III, IV and IX—6.25 μg·L^–1^; V, VI and VII—3.125 μg·L^–1^) on HSS C18 SB stationary phase.
