# Supplementary material for: Supercritical Fluid Chromatography—Tandem Mass Spectrometry for Rapid Quantification of Pentacyclic Triterpenoids in Plant Extracts
Source: Pharmaceuticals (Basel). 2022 May 20;15(5):629. doi: 10.3390/ph15050629 (PMC9143669; doi:10.3390/ph15050629)
Supplement: Supplementary file 1 [file pharmaceuticals-15-00629-s001.zip › pharmaceuticals-1707237-supplementary.pdf]

Supplementary Materials

Supercritical Fluid Chromatography – Tandem Mass Spectrometry for Rapid Quantification of Pentacyclic Triterpenoids in Plant Extracts

Danil I. Falev*, Denis V. Ovchinnikov, Ilya S. Voronov, Anna V. Faleva, Nikolay V. Ul'yanovskii and Dmitry S. Kosyakov*

Laboratory of Natural Compounds Chemistry and Bioanalytics, Core Facility Center "Arktika",
M.V. Lomonosov Northern (Arctic) Federal University, Northern Dvina Emb. 17, 163002 Arkhangelsk, Russia;
* Correspondence: d.falev@narfu.ru (D.I.F.); d.kosyakov@narfu.ru (D.S.K.)

Abstract: Pentacyclic triterpenoids (PCTs) are a widely distributed class of plant secondary metabolites. These compounds have high bioactive properties, primarily antitumor and antioxidant activity. In this study, a method was developed for the quantitative analysis of pentacyclic triterpenoids in plants using supercritical fluid chromatography-tandem mass spectrometry (SFC-MS/MS). Separation of ten major PCTs (betulin, erythrodiol, uvaol, friedelin, lupeol, β-amyrin, α-amyrin, betulinic, oleanolic and ursolic acids) was studied on six silica based reversed stationary phases. The best results (7 min analysis time in isocratic elution mode) were achieved on an HSS C18 SB stationary phase using carbon dioxide – isopropanol (8%) mobile phase providing decisive contribution of polar interactions to the retention of analytes. It was shown that the use of atmospheric pressure chemical ionization (APCI) is preferred over atmospheric pressure photoionization (APPI). The combination of SFC with APCI-MS/MS mass spectrometry allowed for achieving the limits of quantification in plant extracts in the range of 2.3-20 $\mu g \cdot L^{-1}$. The developed method was validated and successfully tested in the analyses of birch outer layer (*Betula pendula*) bark, licorice (*Glycyrrhiza glabra*) root, as well as lingonberry (*Vaccinium vitis-idaea*), cranberry (*Vaccinium oxycoccos*), apple (*Malus domestica "Golden Delicious"* and *Malus domestica "Red Delicious"*) peels.

Keywords: pentacyclic triterpenoids; plant feedstock; supercritical fluid chromatography; tandem mass spectrometry

Table S1. Chromatographic separation parameters of ten PCTs on the stationary phase HSS C18 SB under optimal conditions.

	t_R	k	a	N	Rs
I	2.73	4.25		7455	
II	3.76	6.23	1.47	3194	5.26
III	3.98	6.65	1.07	3579	0.83
IV	4.24	7.15	1.08	3921	0.97
V	4.79	8.21	1.15	10712	2.42
VI	5.15	8.90	1.08	10589	1.87
VII	5.54	9.65	1.08	16142	2.09
VIII	5.76	10.1	1.04	7047	0.98
IX	6.12	10.8	1.07	6298	1.24
X	6.4	11.3	1.05	7180	0.92

Table S2. The results of the evaluation of intraday and inter-day reproducibility analysis of the PCTs on HSS C18 SB stationary phase.

Analyte	Theoretical concentration, μg·L^{-1}	Intra-day assay			Inter-day assay		
		Measured concentration, μg·L^{-1}	Accuracy, %	Precision, %	Measured concentration, μg·L^{-1}	Accuracy, %	Precision, %
I	25	25.0±1.6	100	10.0	27.0±1.3	108	7.68
II	12.5	13.0±0.5	104	5.85	12.8±0.4	103	5.12
III	6.3	6.04±0.32	97	8.50	6.7±0.56	107	13.5
IV	6.3	6.13±0.39	98	10.3	6.88±0.38	110	8.84
V	3.1	3.38±0.15	108	7.10	3.41±0.21	109	9.74
VI	3.1	3.36±0.17	107	8.09	3.27±0.14	105	7.06
VII	3.1	3.16±0.12	101	6.00	3.18±0.17	102	8.56
VIII	12.5	12.5±0.5	100	5.88	13.3±0.8	107	9.45
IX	6.3	7.45±0.25	119	5.42	6.78±0.33	108	7.81
X	12.5	12.2±0.8	97	9.95	12.7±0.6	101	8.20

Table S3. Matrix effect on the determination of PCTs by SFC-APCI-MS/MS estimated by the spike recovery test

Analyte	Initial concentation, μg·L^{-1}	Added, μg·L^{-1}	Found, μg·L^{-1}	Recovery, %
I	-	25	22.1±3.2	88
		200	190±18	95
		2000	2080±80	104
II	536±15	12.5	534±13	97
		100	664±39	104
		1000	1550±30	101
III	357±12	6.25	349±11	96
		50	403±3	99
		500	864±47	101
IV	-	6.25	7.36±2.14	118
		50	47.9±10.1	96
		500	513±43	103
V	149±1	3.125	135±5	89
		25	168±8	97
		250	421±8	106
VI	26.2±2.8	3.125	27.8±7.6	95
		25	49.5±5.4	97
		250	290±13	105
VII	21.3±0.2	3.125	24.2±3.1	99
		25	44.6±1.8	96
		250	262±4	96
VIII	14.2±0.1	12.5	24.6±3	92
		100	108±14	95
		1000	1010±40	100
IX	25.3±0.5	6.25	28.6±3.5	91
		50	68.1±3.4	90
		500	510±18	97
X	-	12.5	12.7±2.4	102
		100	97.4±5.6	97
		1000	991±43	99

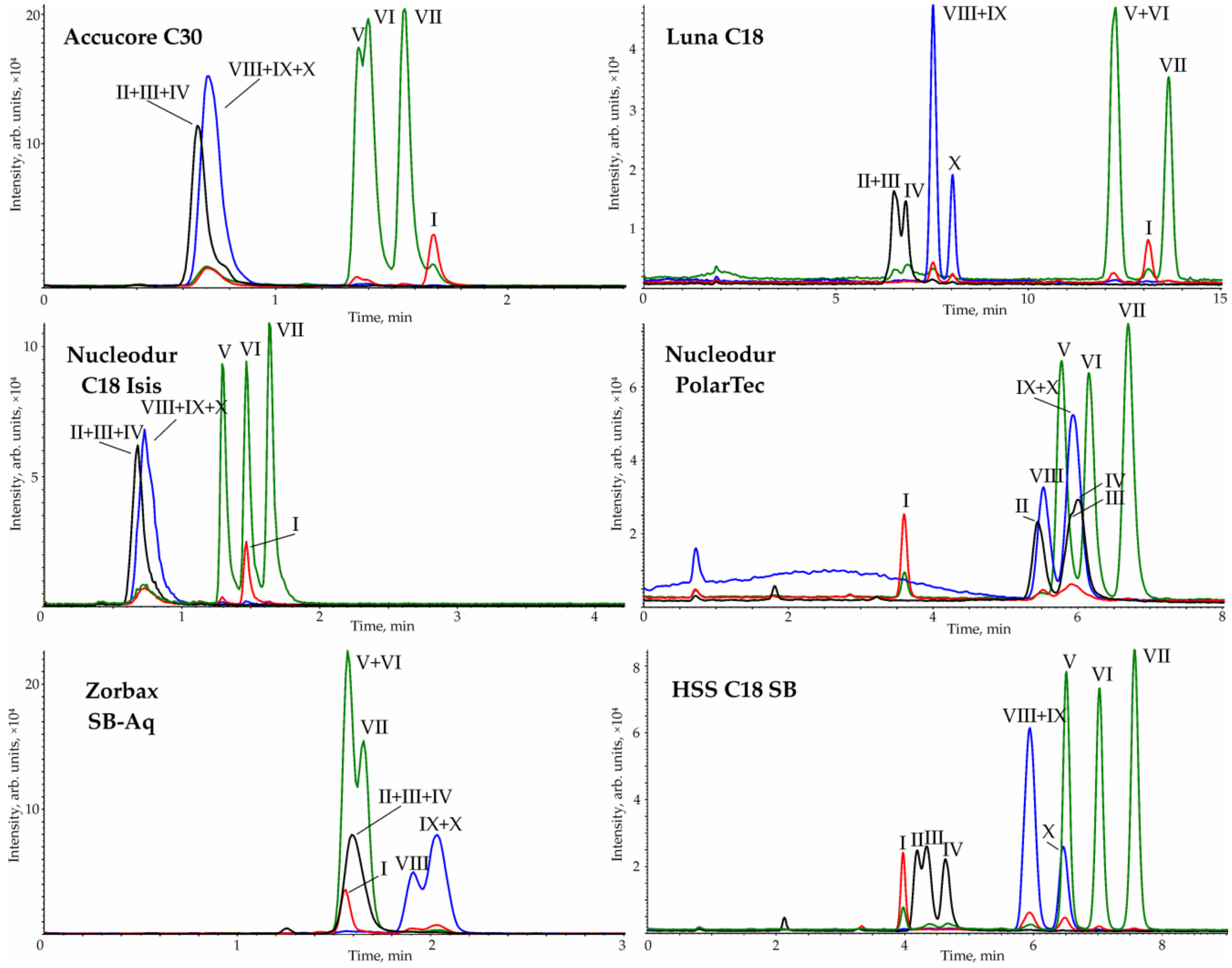

Figure S1. SFC-APCI-MS chromatogram (SIM mode) of analytes model mixture (250 μg·L^{-1} of each compound) obtained on various stationary phases (10% MeOH, 25°C, 150 bar, flowrate 1.0 mL·min^{-1})

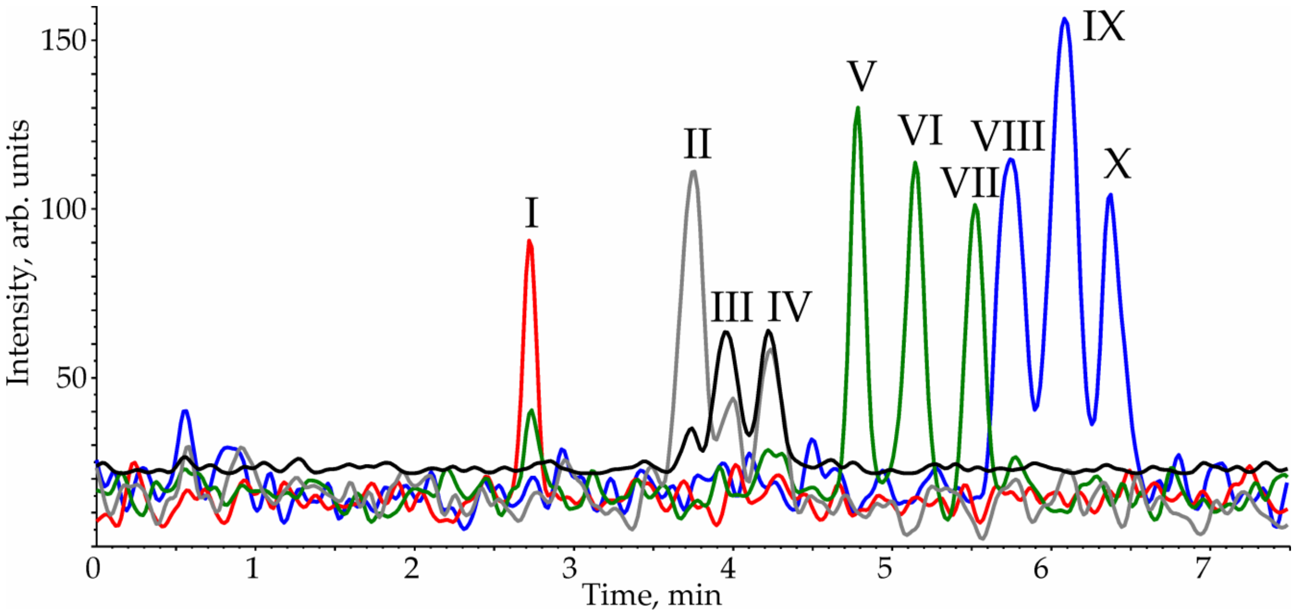

Figure S2. SFC-APCI-MS/MS chromatogram of analytes model mixture (I - 25 μg·L^{-1}; II, VIII and X - 12.5 μg·L^{-1}; III, IV and IX - 6.25 μg·L^{-1}; V, VI and VII - 3.125 μg·L^{-1}) on HSS C18 SB stationary phase (8% iPrOH, 25°C, 150 bar, flowrate 1.5 mL·min^{-1})
